# Total and Regional Brain Volumes in Fetuses With Congenital Heart Disease

**DOI:** 10.1002/jmri.29078

**Published:** 2023-10-17

**Authors:** Daniel Cromb, Alena Uus, Milou P.M. Van Poppel, Johannes K. Steinweg, Alexandra F. Bonthrone, Alessandra Maggioni, Paul Cawley, Alexia Egloff, Vanessa Kyriakopolous, Jacqueline Matthew, Anthony Price, Kuberan Pushparajah, John Simpson, Reza Razavi, Maria DePrez, David Edwards, Jo Hajnal, Mary Rutherford, David F.A. Lloyd, Serena J. Counsell

**Affiliations:** 1 Centre for the Developing Brain, School of Biomedical Engineering and Imaging Sciences, King’s College London, London, UK; 2 Biomedical Engineering Department, School of Biomedical Engineering and Imaging Sciences, King’s College London, London, UK; 3 Department of Cardiovascular Imaging, School of Biomedical Engineering and Imaging Science, King’s College London, London, UK; 4 Paediatric and Fetal Cardiology Department, Evelina London Children’s Hospital, London, UK; 5 MRC Centre for Neurodevelopmental Disorders, King’s College London, London, UK

## Abstract

**Background:**

Congenital heart disease (CHD) is common and is associated with impaired early brain development and neurodevelopmental outcomes, yet the exact mechanisms underlying these associations are unclear.

**Purpose:**

To utilize MRI data from a cohort of fetuses with CHD as well as typically developing fetuses to test the hypothesis that expected cerebral substrate delivery is associated with total and regional fetal brain volumes.

**Study Type:**

Retrospective case−control study.

**Population:**

Three hundred eighty fetuses (188 male), comprising 45 healthy controls and 335 with isolated CHD, scanned between 29 and 37 weeks gestation. Fetuses with CHD were assigned into one of four groups based on expected cerebral substrate delivery.

**Field Strength/Sequence:**

T2-weighted single-shot fast-spin-echo sequences and a balanced steady-state free precession gradient echo sequence were obtained on a 1.5 T scanner.

**Assessment:**

Images were motion-corrected and reconstructed using an automated slice-to-volume registration reconstruction technique, before undergoing segmentation using an automated pipeline and convolutional neural network that had undergone semi-supervised training. Differences in total, regional brain (cortical gray matter, white matter, deep gray matter, cerebellum, and brainstem) and brain:body volumes were compared between groups.

**Statistical Tests:**

ANOVA was used to test for differences in brain volumes between groups, after accounting for sex and gestational age at scan. *P*_FDR_-values <0.05 were considered statistically significant.

**Results:**

Total and regional brain volumes were smaller in fetuses where cerebral substrate delivery is reduced. No significant differences were observed in total or regional brain volumes between control fetuses and fetuses with CHD but normal cerebral substrate delivery (all *P*_FDR_ > 0.12). Severely reduced cerebral substrate delivery is associated with lower brain:body volume ratios.

**Data Conclusion:**

Total and regional brain volumes are smaller in fetuses with CHD where there is a reduction in cerebral substrate delivery, but not in those where cerebral substrate delivery is expected to be normal.

**Evidence Level:**

3 **Technical Efficacy:** Stage: 3

**C**ongenital heart disease (CHD) is the most common group of congenital malformations, affecting ∼1% of live births.^1^ CHD is also associated with developmental impairments across multiple domains including speech and language, motor coordination and cognition.^2^

The mechanisms which underlie these impairments remain unclear, but are likely to be multifactorial, with periods of postnatal hypoxia, the surgery and associated pro-longed hospital stays all implicated.^3,4^ Notably, MRI studies have shown that brain development in infants with CHD is abnormal prior to surgery, including an increased risk of brain injury and reduced global and regional volumes.^5–8^

There is mounting evidence that CHD is associated with impaired brain development in-utero, with multiple studies identifying smaller total^9–11^ and regional^12,13^ brain volumes in different types of CHD.^14–17^ Underlying cardiac physiology and subsequent cerebrovascular hemodynamics play an important role, particularly in the presence of reduced cerebral oxygenation.^18–20^

In the normal fetus, inter-atrial and systemic arterial shunts are present, with both the right and left sides of the heart supporting the fetal circulation in parallel. A notable feature of the normal fetal circulation is the preferential streaming of oxygen and nutrient rich placental blood to the fetal brain. The umbilical vein enters the fetus via the umbilicus, and after a portion then drains to the portal circulation, the remainder is shunted to the right heart via the ductus venosus. This blood is then preferentially streamed across the patent foramen ovale by the Eustachian valve into the left atrium, left ventricle and ultimately to the developing fetal brain.^21^

The effects of CHD on the substrate content, including oxygen, glucose, and other nutrients, of blood reaching the fetal brain have been categorized in various ways, based on both directly measured MRI data, and the theoretical impacts based on underlying anatomy.^18,22,23^ Different types of CHD will impact this streaming pattern in different ways, with potentially important implications for the delivery of substrate-rich blood to the developing brain.^11,19,24^ For example, in some conditions, such as a right aortic arch or a double aortic arch, there is no theoretical disruption either to intracardiac streaming or the vascular pathway of streamed blood from heart to the brain.^25^ In fetuses with suspected coarctation of the aorta (CoA), a cardinal sign is flow reversal at the aortic isthmus,^26^ which, in the absence of other major lesions will result in a variable amount of fetal systemic venous blood being redirected back to the cerebral circulation. In lesions where there is only one functional outlet to the heart, such as truncus arteriosus, pulmonary atresia or hypoplastic left heart syndrome, placental and fetal systemic venous blood are necessarily mixed before leaving the heart, and the substrate availability of blood reaching the brain is further reduced.^19^ In transposition of the great arteries (TGA), the two main arteries leaving the heart—the pulmonary artery and the aorta—arise from the left and right ventricles respectively, leading to a complete reversal of normal fetal streaming patterns, with systemic venous return comprising the majority of cerebral blood flow.^19^

Imaging the fetal brain remains challenging. Fetal and maternal motion can cause image artifacts. The fetal brain is small, and some structures such as the cortex, are only a few millimeters thick. As such, high-resolution imaging is required to delineate brain structures posing challenges for signal-to-noise ratio (SNR). Recent advances in MRI acquisition and reconstruction techniques enable sub-millimeter resolution imaging of the fetal brain that is resistant to fetal motion.^27^ This allows characterization of early brain volumetric development in both typically developing fetuses and those with pathology affecting brain growth, including CHD.

Previous studies have focused on individual cardiac diagnoses, such as TGA or HLHS, and sometimes treat all types of CHD as a heterogeneous group.^9,10,14,28^ Additionally, previous work has focused largely on CHD lesions defined as critical or severe, requiring early postnatal intervention, with other less severe forms of CHD less well described.^10,14,29–31^ When considering brain development in CHD, a physiological rather than diagnostic approach may be beneficial, particularly with the potential benefits this affords when it comes to understanding the biological mechanisms behind why brain growth may be impaired in an individual fetus with CHD.

The aim of this study was to utilize advanced fetal neuroimaging data from a large cohort of fetuses with suspected or confirmed CHD as well as typically developing fetuses to test the hypothesis that expected cerebral substrate delivery in-utero is associated with total and regional volumes in the fetal brain.

## Materials and Methods

### Ethical Approval

The National Research Ethics Service West London committee provided ethical approval (07/H0707/105; 14/LO/1806; 17/LO/0292). Informed written consent was obtained before fetal MRI.

### Subjects

For this retrospective study, all imaging data was acquired between June 2014 and June 2022. For the CHD cohort, 529 women (age at scan = 31.52 [±5.73] years) carrying a fetus with known or suspected CHD who consented for research prior to clinical fetal cardiac MRI scans, or from research MRI scans where appropriate consents were already in place for analysis of these data. For the control cohort, pregnant women undergoing fetal MRI beyond 29 weeks gestation who consented to the images being used for research were included. For both cohorts, maternal exclusion criteria included multiple pregnancy, weight over 125 kg, severe claustrophobia, inability to give informed consent, or age under 18 years at the time of referral. Fetal exclusion criteria included: Confirmed genetic abnormalities on antenatal or postnatal genetic testing (either from amniocentesis, comparative genomic hybridization array [CGH-array], or clinical genetic testing); major fetal extracardiac anomalies, such as congenital diaphragmatic hernia or duodenal atresia; or structural brain abnormalities as reported on fetal MRI, including bilateral ventriculomegaly, cerebellar hypoplasia or absence of the corpus callosum.

#### Mr Image Acquisition

Fetal MR images were acquired on a Philips Ingenia 1.5 T scanner, with 28-channel dStream anterior and posterior in-built coils. Fetal brain images were acquired using a 2D T2-weighted fast-spin-echo sequence (Repetition Time [TR] = 15,000 msec, Echo Time [TE] = 80 msec, voxel size = 1.25 × 1.25, slice thickness 2.5 mm, slice spacing 1.25 mm, field-of-view = 420 × 420 mm, matrix = 336 × 336, SENSE factor = 2, flip angle 90°, bandwidth = 413 Hz), optimized for fetal imaging.^32^ To image the fetal body, sagittal whole-uterus images were acquired using a balanced steady-state free precession (bSSFP) sequence (TR = 5 msec, TE = 1.98 msec, voxel size = 1.25 × 1.25, slice thickness 5 mm, field-of-view = 420 × 420 mm, matrix = 288 × 288, SENSE factor = 2, flip angle = 90^R0^, bandwidth = 689 Hz). No sedation or intravenous contrast was used.

#### Fetal Brain Image Reconstruction

**Figure 1:**
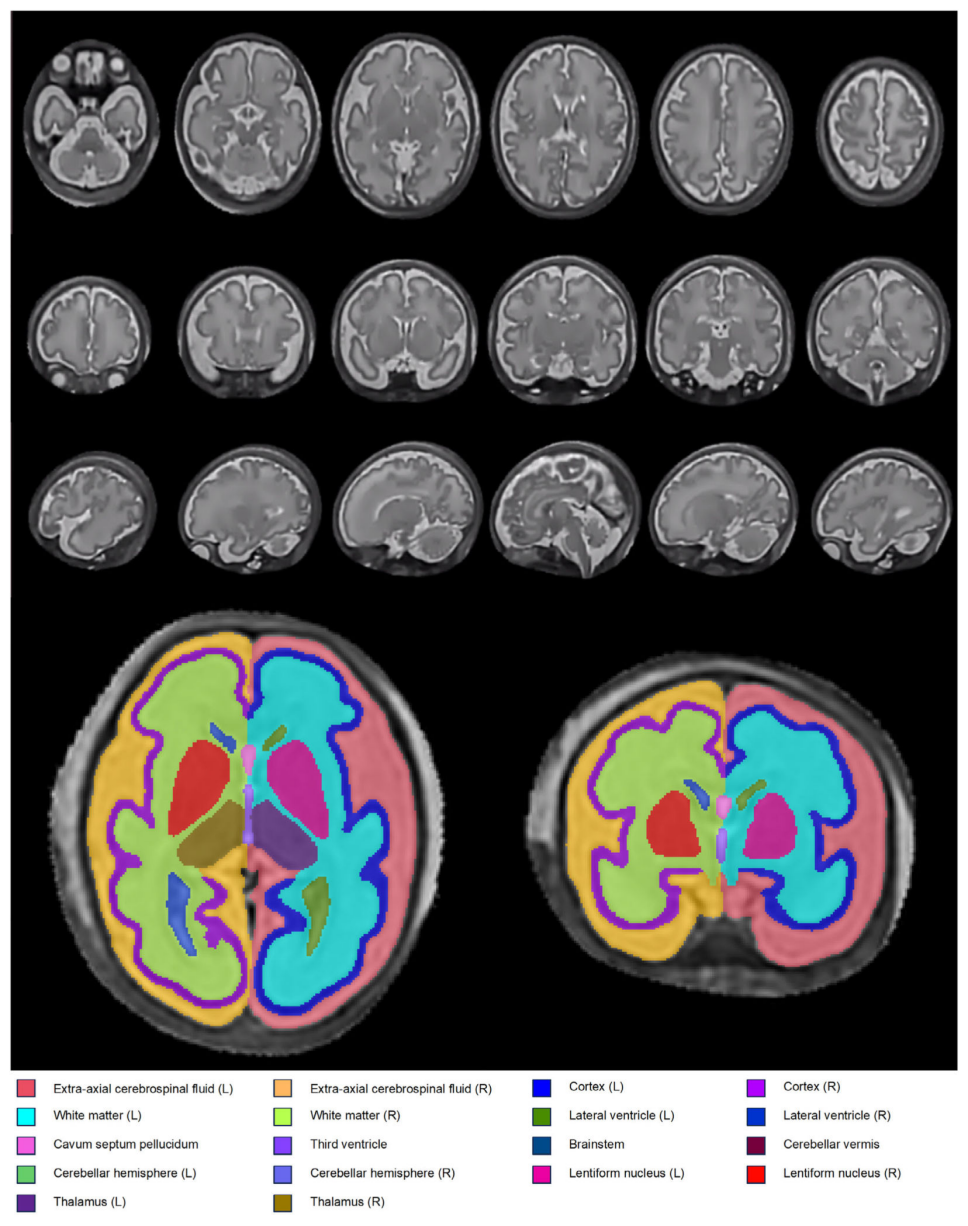
Example 3D slice-to-volume reconstructions of T2-weighted fetal brain MR images in coronal (first row), axial (second row), and sagittal (third row) views. These images were acquired at a gestational age of 34^+0^ weeks. Regional segmentations, based on the developing Human Connectome Project atlas segmentation protocol with 19 label regions of interest, generated by BOUNTI automated fetal brain MRI segmentation tool are shown in axial (bottom left) and coronal (bottom right) slices.

Images were motion corrected and reconstructed using an automated version of the classical 3D slice-to-volume registration (SVR) reconstruction technique,^33,34^ (auto brain reconstruction SVRTK docker—https://github.com/SVRTK/SVRTK; https://hub.docker.com/r/fetalsvrtk/svrtk tag *auto-2.20*), before being up sampled to 0.5 mm^3^ isotropic resolution (Fig. 1).

#### Image Review

MR images were reported by perinatal neurora-diologists (MR, AE), both with over 10 years experience. The presence of any structural abnormalities was recorded. The quality of the fetal brain reconstructions, taking into account the definition of anatomical features, signal to noise ratio, contrast and alignment, was independently assessed by two clinicians (DC, PC) trained in the assessment of fetal and neonatal brain MRI (DC = 5 years experience, PC = 4 years experience), with each image being graded as good (4/4), acceptable (3/4), poor (2/4) or failed (1/4), as previously described.^35^ Only datasets where both reviewers gave a rating of acceptable or good were included. An example of a good quality reconstruction is shown in Fig. 1.

### Fetal Brain Segmentation

Each brain SVR was segmented using an automated *Brain vOlumetry and aUtomated parcellatioN for 3D feTal MRI* (*BOUNTI*) pipeline^36^ (SVRTK segmentation docker: https://hub.docker.com/r/fetalsvrtk/segmentation tag *bounti_brain_tissue_1.00*) based on the developing Human Connectome Project atlas segmentation protocol with 19 label regions of interest (https://gin.g-node.org/kcl_cdb/fetal_brain_mri_atlas) and a combination of 3D UNet and Attention UNet^37^ convolutional neural networks that had undergone semi-supervised training in the *Medical Open Network for Artificial Intelligence* (MONAI). All resulting segmentations were reviewed and failed or inaccurate segmentations were excluded. Those confirmed to be acceptable for volumetry were kept. No manual editing was performed on segmentations.

**Figure 2:**
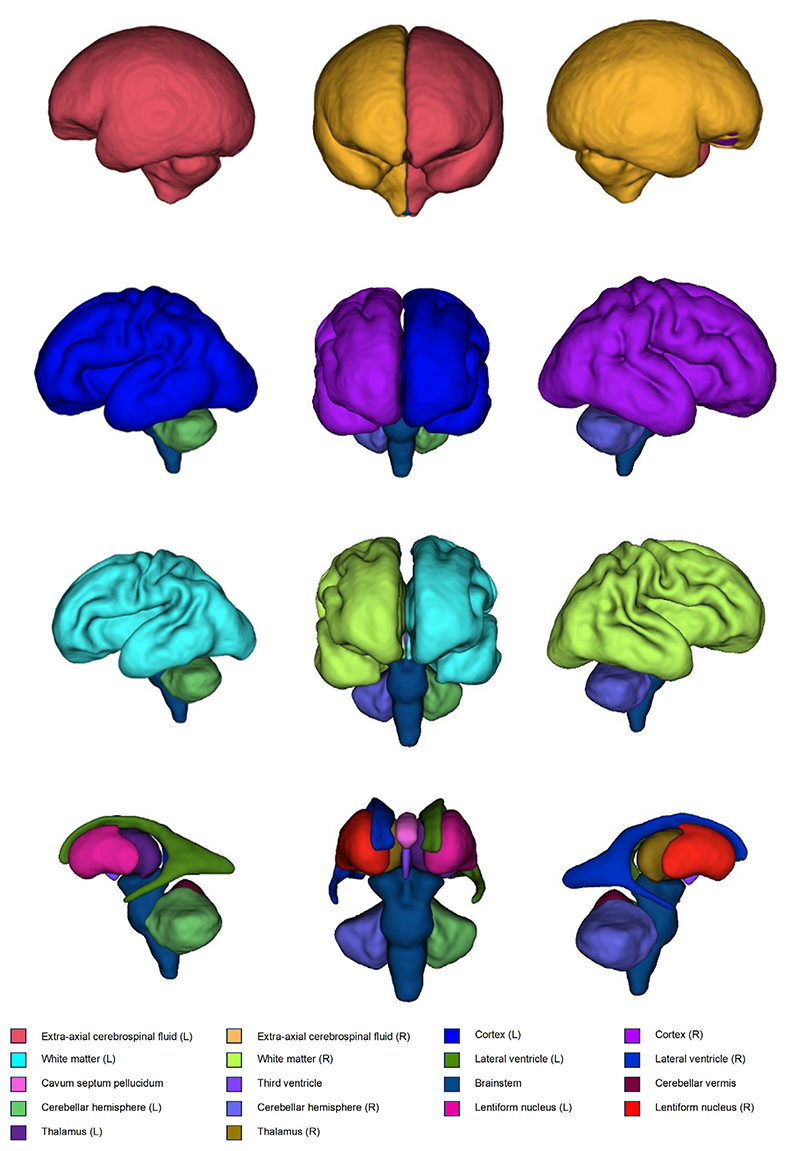
3D representations of fetal brain MRI segmentations, for a fetus imaged at 34^+0^ weeks, from which total and regional volumes were generated.

Volumes were generated for five brain tissue regions: 1) cortical gray matter (cGM); 2) white matter (WM); 3) deep gray matter (dGM) (thalamus + lentiform nucleus); 4) cerebellum (cerebellar hemispheres + cerebellar vermis); and 5) brainstem. Volumes were also calculated for total cerebrospinal fluid (CSF) by summing the volume of extra-axial CSF, lateral ventricles, cavum septum pellucidum, and the third fourth ventricles. Total brain tissue volume was calculated by summing cGM, WM, dGM, cerebellum, and brainstem. These segmentations and their volumetric representations can be seen in Figs. 1 and 2.

### Fetal Body Segmentation

**Figure 3:**
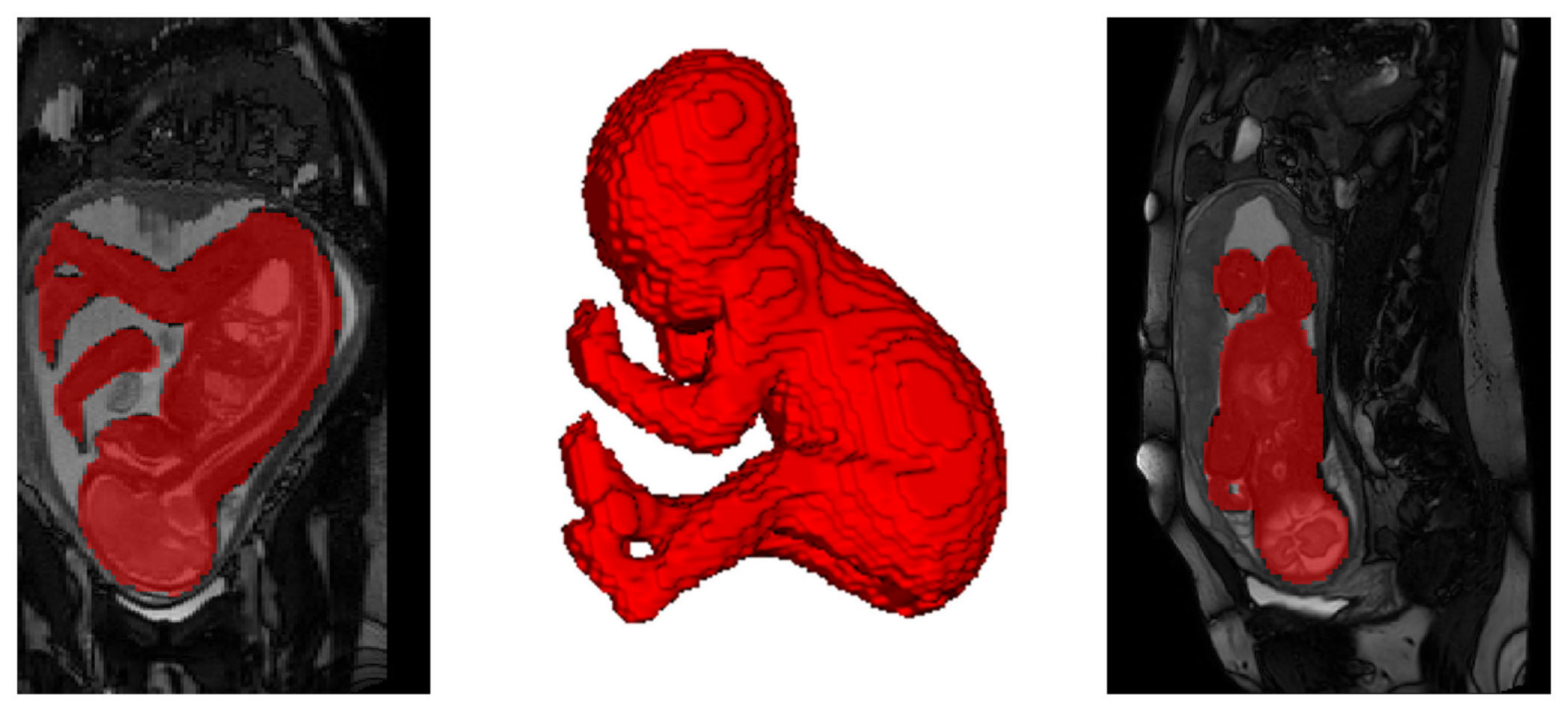
Automatically generated whole-fetus segmentations, allowing estimation of total fetal body volumes, from T2-weighted balanced steady-state free precession (bSSFP) sequences. Coronal bSSFP + segmentation (left), 3D fetal body rendering (middle), Sagittal bSSFP + segmentation (right).

A separate 3D UNet^37^ convolutional neural network that had undergone semi-supervised training in MONAI was used to generate fetal whole-body segmentations, using the sagittal whole-uterus T2-weighted bSSFP images (SVRTK segmentation docker: https://hub.docker.com/r/fetalsvrtk/segmentation tag *btfe_whole_body*). The whole-uterus images and resulting segmentations were independently assessed for motion artifacts and whether there was any cropping of the fetal body (by DC, 5 years experience & MVP, 6 years experience). Datasets with minimal motion artifact, no cropping of the region of interest, and fetal body segmentations considered good quality were included (Fig. 3). Estimated fetal body volumes were derived from these whole-body segmentations using ITK-snap.^38^

### Fetal CHD Categorization

In the absence of direct measurements of cerebral substrate delivery in this cohort, cases were classified according to the expected effect of the underlying cardiac defect, using MRI derived fetal blood flows where available, as described in Lloyd et al,^26^ on the oxygen and nutritional content of blood delivered to the carotid arteries, and therefore the brain (referred to onwards for brevity as “cerebral substrate content”). Fetal echocardiogram data were acquired as per routine clinical care and where information about the direction of blood flow at the aortic isthmus was important for CHD categorization, and this information was not otherwise available from MRI derived fetal blood flows, it was extracted from the clinical fetal echocardiogram report. Other speculative effects on streaming, abnormal flow patterns or determination of specific substrates were not included. This led to the development of four distinct categories:

1. Substrate content of cerebral blood is expected to be normal;
2. Substrate content of cerebral blood is expected to be mildly reduced (i.e., some mixing of placental and fetal systemic venous blood);
3. Substrate content of cerebral blood is expected to be moderately reduced (i.e., complete mixing of placental and fetal systemic venous blood);
4. Substrate content of cerebral blood is expected to be severely reduced (i.e., complete reversal of normal placental streaming).

In cases where a diagnosis could potentially fit into more than one category, depending on severity or underlying hemodynamics, a combination of phase contrast (with metric optimized gating) fetal flow measurements (described in Refs.^26,39^) and/or contemporaneously acquired echocardiographic data were used to assign cases individually, following assessment of the data by a fetal cardiologist.

A summary of all diagnoses, including the rationale for inclusion in the categories above, is given in Table S1 in the Supplemental Material.

### Statistical Analyses

All statistical analyses were performed using statsmodels (v0.13.2) and Jupyter Notebook, python3. A one-way ANOVA was used to test for differences in volumes between groups, after accounting for relevant confounders. We first performed an analysis comparing total and regional brain volumes in the “control” group of typically developing fetuses (Group 0) and fetuses with CHD where cerebral substrate delivery is expected to be normal (Group 1), after accounting for GA at scan and fetal sex. We then performed an analysis comparing total and regional brain volumes between groups for all fetuses in the study. For this analysis, both typically developing fetuses and fetuses with CHD where cerebral substrate delivery is expected to be normal were assigned to the same group, with the label “normal cerebral substrate delivery.” We also ran an additional post-hoc analysis to explore whether there were any differences in fetal brain:body ratios between each of the groups described above, after accounting for GA at scan and fetal sex. Benjamini and Hochberg false discovery rate (FDR) was applied to correct for multiple comparisons (reported as *P*_FDR_). *P*_FDR_-values <0.05 were considered statistically significant.

## Results

### Demographics

**Figure 4:**
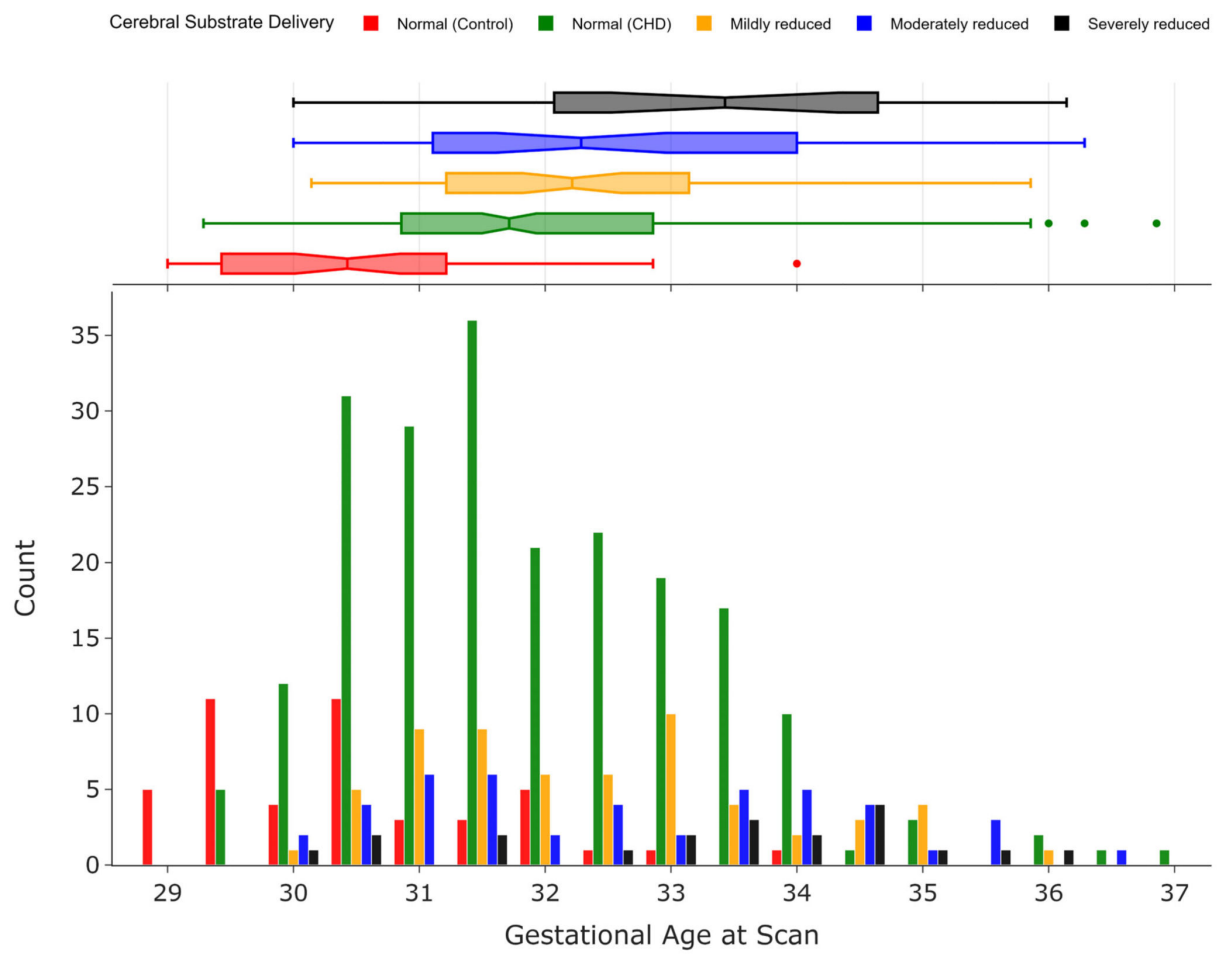
Histograms showing counts for gestational age at the time of MRI scan for all 380 fetuses (192 female, 188 male) included in this study, classified according to their expected cerebral substrate delivery.

Data from 380 fetuses me the inclusion criteria: 45 healthy controls and 335 with CHD. A breakdown of the number of fetuses allocated to each of the different groups based on expected cerebral oxygenation and the median (IQR) age at scan for each group is shown in Table 1. There were 192 female fetuses and 188 males. There was a significant difference in GA at scan (), but not fetal sex (*F* = 2.01, *P* = 0.28) between groups. Histograms showing the distribution of GA at scan for each group are shown in Fig. 4.

### CHD With Expected Normal Cerebral Substrate Delivery Compared to Controls

**Figure 5:**
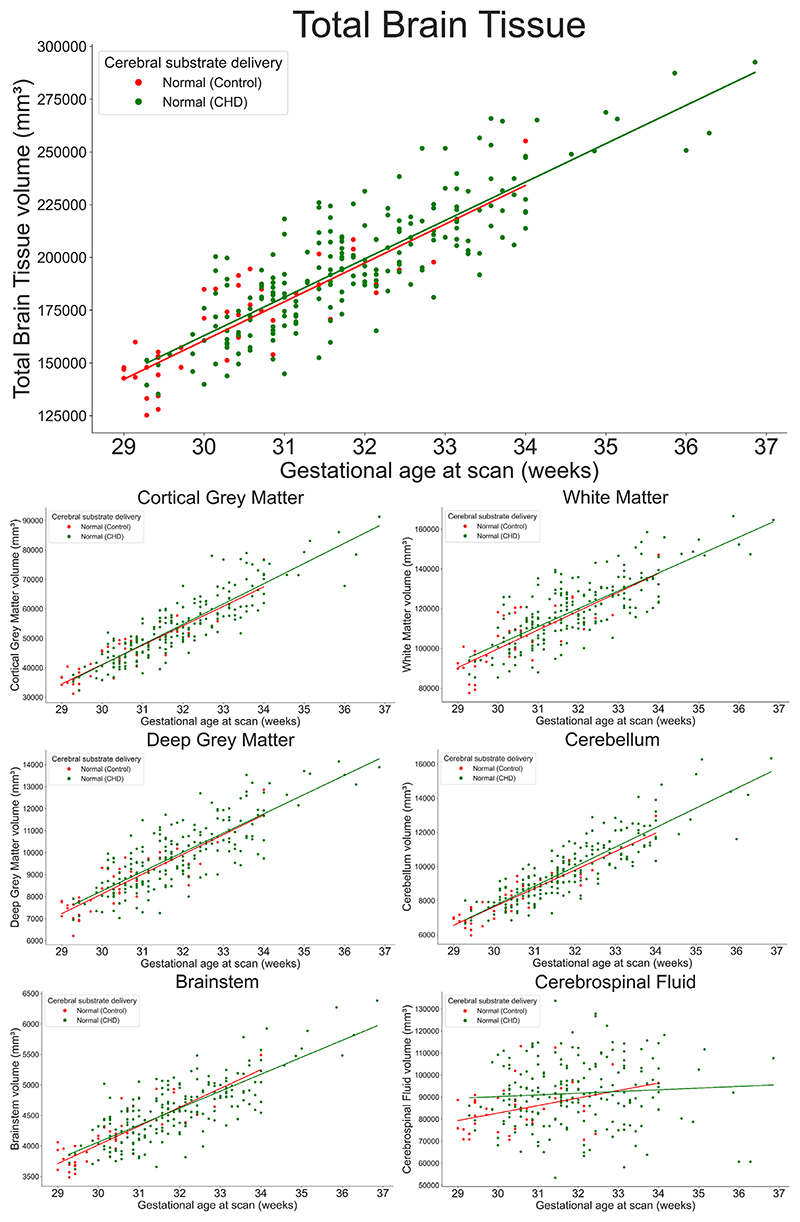
Scatter plots showing total brain tissue (top panel) and regional brain volumes across gestational age for 45 typically developing fetuses and 210 fetuses with CHD where cerebral substrate delivery is expected to be normal. After accounting for sex and gestational age at scan, no significant group difference was seen in any brain tissue regions or CSF volumes (All *P*_FDR_ > 0.12) between these two groups.

There was a significant difference in GA at scan, but not fetal sex (*F* = 0.27, *P* = 0.60) between the control group of typically developing fetuses (Group 0) and fetuses with CHD where cerebral substrate delivery is expected to be normal (Group 1). After accounting for sex and GA at scan, no significant difference was seen in total brain tissue, or any regional brain tissue or CSF volumes between these two groups (All *P*_FDR_ > 0.12) (Fig. 5).

### Differences in Total and Regional Brain Volumes Between Groups

**Figure 6:**
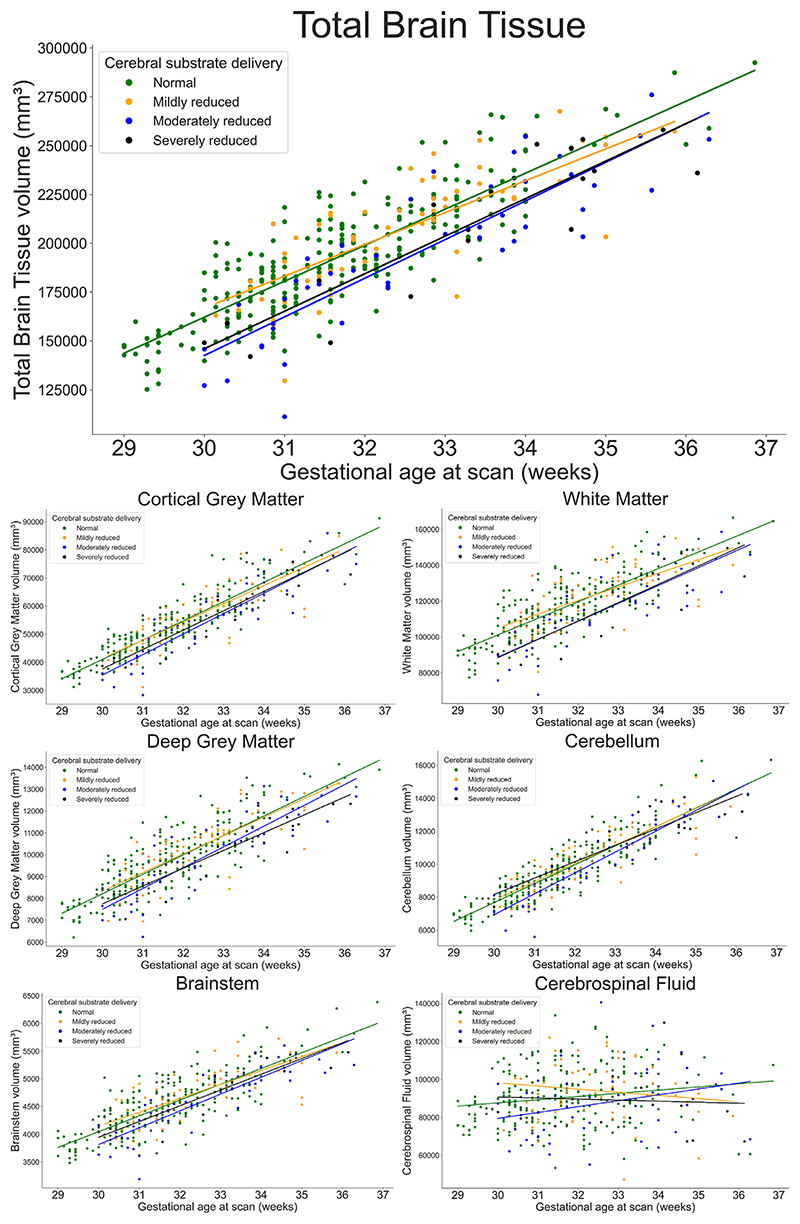
Scatter plots showing total brain tissue (top panel) and regional brain volumes across gestational age for fetuses grouped according to expected cerebral substrate delivery for 380 fetuses. There is a significant difference in total brain tissue (*P*_FDR_ = 4.8 × 10^−8^), cortical gray matter (top left; *P*_FDR_ = 8.3 × 10^−6^), white matter (top right; *P*_FDR_ = 1.19 × 10^−8^), deep gray matter (middle left; *P*_FDR_ = 3.7 × 10^−5^), cerebellum (middle right; *P*_FDR_ = 3.44 × 10^−2^), and brainstem (bottom left; *P*_FDR_ = 4.0 × 10^−3^) volumes between groups after accounting for sex and gestational age at scan, but not cerebrospinal fluid volumes (bottom right; *P*_FDR_ = 0.13).

After accounting for sex and gestational age at scan, there was a significant difference in total brain tissue volume and cGM, WM, dGM, cerebellum and brainstem volumes (between all four groups, but not in CSF volumes (*P*_FDR_ = 0.13) (Fig. 6). These reductions in brain volumes are evident in-utero from 30 weeks gestation and persist until at least 37 weeks gestation.

Post-hoc analyses showed that, after accounting for sex and gestational age at scan, there was a significant difference in total brain tissue volumes between fetuses with normal cerebral substrate delivery and both moderately reduced cerebral substrate delivery () and severely reduced cerebral sub-strate delivery (), but not between fetuses with normal cerebral substrate delivery and mildly reduced cerebral sub-strate delivery (*F* = 0.43, *P* = 0.47) or between fetuses with moderately and severely reduced cerebral substrate delivery (*F* = 0.11, *P* = 0.53).

### Fetal Brain:Body Volume Ratios

Paired fetal brain and body segmentations were available for 256 of the 380 fetuses described above (134 female, 122 male. Substrate content of cerebral blood is expected to be normal [Group 1], N = 183; Substrate content of cerebral blood is expected to be mildly reduced [Group 2], N = 39; Substrate content of cerebral blood is expected to be moderately reduced [Group 3], N = 20; Substrate content of cerebral blood is expected to be severely reduced [Group 4], N = 14). There remained a significant difference in gestational age at scan () but not fetal sex (*P* = 0.16) between groups.

**Figure 7:**
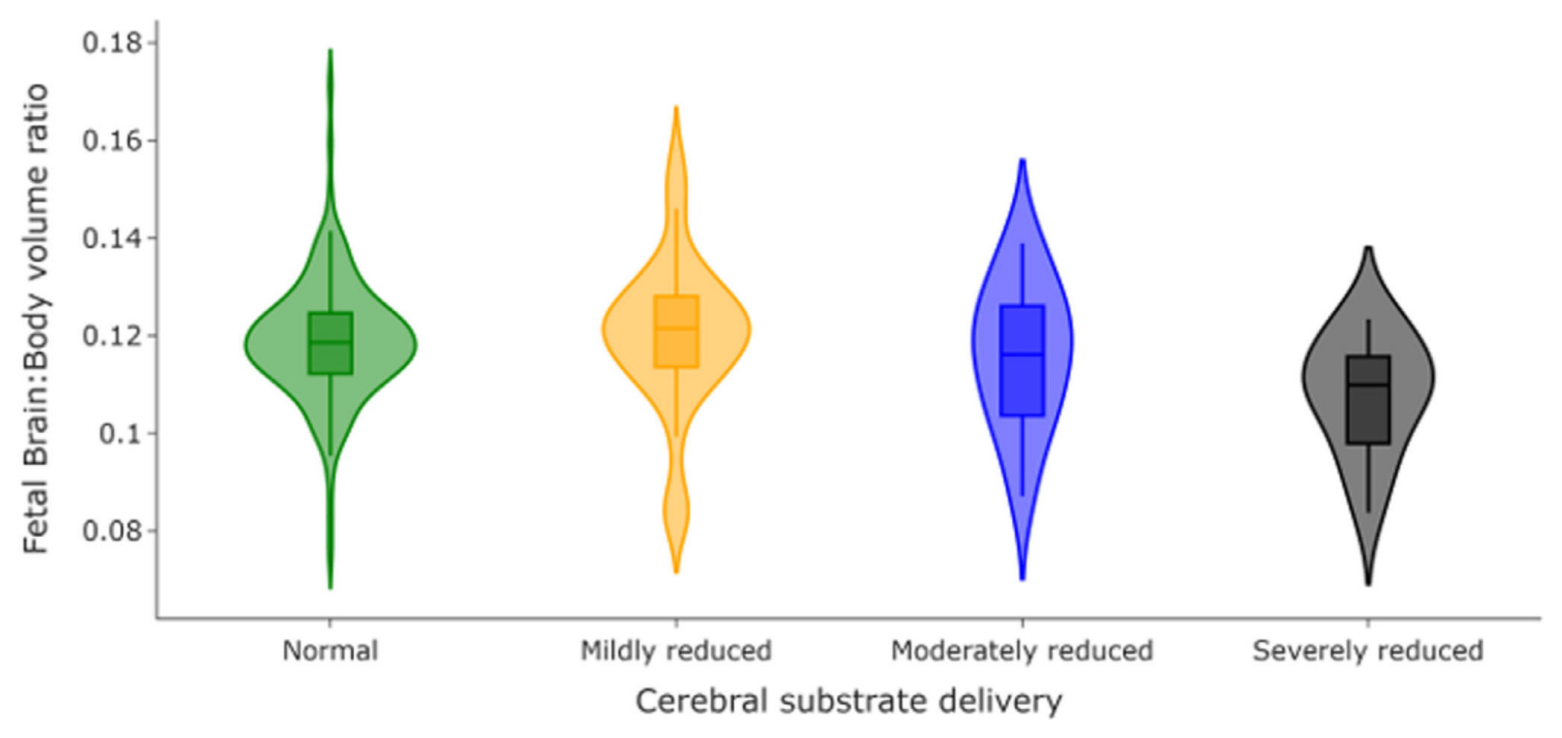
Violin plots comparing brain:body volume ratios for 256 fetuses, grouped according to expected cerebral substrate delivery. Fetuses with severely reduced cerebral substrate delivery have significantly lower brain:body ratios than those with normal cerebral substrate delivery (*P*_FDR_ = 0.004).

After accounting for sex and gestational age at scan, there was no significant difference in total body volume between groups (*F* = 1.18, *P* = 0.32), but there was a significant difference in fetal brain:body volume ratios (). A further subsequent post-hoc analysis showed that there was no significant difference in fetal brain:body ratios between fetuses with normal cerebral substrate delivery and either fetuses with mildly reduced cerebral substrate delivery (*F* = 0.23, *P*_FDR_ = 0.47) or with moderately reduced cerebral substrate delivery (*F* = 0.95, *P*_FDR_ = 0.392) but there was a significant difference in fetal brain:body ratios between fetuses with normal cerebral substrate delivery and fetuses with severely reduced cerebral substrate delivery () (Fig. 7).

## Discussion

This study utilized automated MR image reconstruction and segmentation techniques to generate sub-millimeter resolution, motion-tolerant fetal brain images with corresponding segmentations of multiple brain tissue regions to show that fetal cardiac physiology resulting in a presumed reduction in expected cerebral substrate delivery is associated with significantly reduced total and regional brain volumes in cortical gray matter, white matter, deep gray matter, the cerebellum and the brainstem.

This work aligns with recent research showing that reduced cerebral oxygenation, as measured using T2-relaxometry, is associated with smaller brain volumes in fetuses with CHD.^20^ It also has important implications for understanding the impaired neurodevelopmental outcomes seen in individuals with CHD, given the associations identified between fetal brain volumes and subsequent neurodevelopmental outcomes in this population.^40^

The approach to categorizing CHD differs from previously published studies in that CHD groups were classified according solely to the expected effect of the underlying cardiac defect on the streaming of substrate-rich placental blood to the cerebral circulation, supported by fetal phase-contrast MRI flow data and/or contemporaneous echocardiography where available. It is important to note disordered streaming is one of several proposed mechanisms affecting substrate delivery and/or brain development in fetuses with CHD. For example, as described in Rudolph,^41^ “in aortic atresia blood flow to the brain is derived from the right ventricle, passing circuitously through the pulmonary artery, ductus arteriosus, and aortic arch. In addition, CoA frequently imposes some obstruction to flow into the aortic arch. In pulmonary atresia, the left ventricle ejects a large stroke volume directly into the ascending aorta.” Therefore, even when the oxygenation of blood reaching the brain is the same in different diagnoses, blood flow patterns, pulsatility and pressures, and the availability of other substrates besides oxygen (i.e., glucose availability) are all likely to play a role.^41,42^ Recent MRI advances that have allowed in-utero quantification of arterial and venous oxygen saturations in fetal blood vessels,^19,23,43^ as well as visualization of 4D flow patterns in the heart and major vessels,^22^ may in future allow for a more detailed analysis of the relationship between the fetoplacental circulation and prenatal brain development in CHD.

We included fetuses with suspected coarctation prenatally who were not shown to have this condition in the neonatal period (“false positive” or CoA (–)). It has been shown previously that this group differs significantly from a healthy control population in terms of the distribution of the fetal circulation, for reasons that remain unclear.^26^ Arguably they represent a unique group in this cohort as they do not have postnatally confirmed CHD, however, as all cases were referred prenatally, this outcome was not known at the time of fetal imaging, and MRI flow and/or ultrasound data were available to make informed per case estimates regarding cerebral blood oxygen content. As the primary hypothesis of this work is that reduction in cerebral substrate delivery negatively impacts cerebral growth, and data were available to stratify this for the CoA (–) group, this group was retained for the analysis.

These results support existing literature on altered volumetric brain development in fetuses with CHD. They show that total brain volumes are reduced in CHD associated with an expected reduction in cerebral substrate.^9,10,44^ In line with previous work looking at specific individual brain regions, the results also show that growth is impaired in the cerebellum and brainstem,^13^ cortical gray matter and white matter.^14^ Imaging was all performed in the third trimester of pregnancy and thus complements existing work exploring associations between reduced cerebral substrate delivery and brain volumes in earlier gestation.^11,17^

After accounting for fetal sex and gestational age at scan, there was no significant difference in total brain tissue volumes between fetuses where cerebral substrate delivery is expected to be moderately and severely reduced, as highlighted. While this work suggests that the substrate content of blood reaching the fetal brain is an important factor in determining brain growth, this particular result highlights that there are other important variables that impact cerebral substrate delivery in-utero and should be considered, such as measures of placental function or additional environmental factors.^45^

For the group with normal cerebral substrate delivery, representing the largest individual group in this study, no significant difference in total or regional brain volumes between these fetuses and a cohort of typically developing fetuses was identified (), supporting the hypothesis that cerebral substrate delivery is a key mediator of fetal brain growth in CHD. Knowing that volumetric brain development is less likely to be affected in specific subtypes of CHD may be helpful for clinicians to identify appropriate groups for neurodevelopmental follow-up and support in postnatal life, as well as focusing future research efforts in this area.

Recent work has also highlighted that reduced fetal brain volumes in CHD are associated with an increased risk of subsequent neonatal ischemic injury^13^ and neonatal white matter injury.^46^ Linking reduced fetal brain volumes with expected cerebral substrate delivery in CHD allows speculation as to the underlying biological mechanisms involved in these processes,^41,47^ and provides further incentives to study fetal brain volumes in fetuses with CHD.

The results also indicate that fetal brain:body volumes are significantly reduced in fetuses with reduced cerebral substrate delivery in-utero, suggesting that these fetuses not only have smaller absolute brain volumes, but smaller brain volumes than would be expected when considering the size of their body. Fetal brain development is a complex, tightly regulated process, but reduced oxygen levels, however they occur, can disrupt these processes, leading to overall diminished brain growth.^20,48,49^ Potential “brain-sparing” compensatory mechanisms such as cerebrovascular dilatation, increased cardiac output or hematological adaptations can mitigate some of these effects.^50^ However, these compensatory mechanisms are likely to rely at least in part on normal streaming of oxygen-rich blood from the placenta. It is interesting to note therefore that when this fetal streaming is entirely reversed in-utero, resulting in a severe reduction in the substrate content of cerebral blood, as seen in fetuses with TGA, there is a greater-than-expected reduction in brain growth, whereas body growth remains relatively unaffected. This finding agrees with previous studies showing disproportion in head circumference and birth weight in fetuses and newborns with TGA.^10,51^ This brain:body volume relationship in TGA fetuses may be an important consideration in interpreting both indexed flow measurements and normal growth trajectories in utero.

### Limitations

This study was performed in a single center, using a single magnet, vendor and field strength (1.5 T), which may limit how widely the results can be interpreted.

This work involves making some assumptions about expected cerebral oxygen and substrate delivery based on the underlying cardiac physiology, supported by additional fetal imaging such as fetal echocardiography or MRI-derived cardiac flow data. While there is work that quantifies actual arterial and venous oxygen saturations in major blood vessels in fetuses with and without CHD,^22,23,43^ it is not possible to know if the assumptions made are true for each individual fetus. In addition, other factors that might be relevant in the altered cerebrovascular hemodynamics that is seen in the fetus with CHD, such as whether the blood reaching the brain is arriving under normal pressures, whether the blood from the brain drains normally or what the likely impact on the altered cardiac physiology is on cerebrovascular resistance were not explored. These considerations, coupled with the innovative methods described above, allowing in-utero quantification of oxygen saturations in fetal blood vessels, represent important directions for future research in this area.

This work focuses on fetuses with suspected or confirmed CHD imaged between 29- and 37-weeks gestational age. This reflects current clinical practice, mainly related to the optimal timing of fetal cardiac MR imaging following initial diagnosis, taking into account referral time, the gestational age at diagnosis and the potential impact of new information on fetal counseling and management that may be required. Linear growth for total and regional brain volumes during these gestational ages was assumed, in-line with other literature.^11^ Future advanced imaging studies with longitudinal data in pregnancy may help to understand both how and when these changes in brain growth occur.

Finally, all fetuses with some degree of flow reversal in the aortic arch were classified into Group 2 (oxygen content of cerebral blood is expected to be mildly reduced). This group represents a range of conditions where blood flow into the carotid arteries up to the fetal brain cannot be safely assumed to be normal. However, this may be a relatively minor effect in some cases, which may in part explain why no significant difference in total brain tissue volumes between fetuses in Group 1 and Group 2 were identified. Future studies leveraging non-invasive MRI methods as above to quantify cerebral oxygen delivery more accurately, may help to better define the thresholds beyond which fetal brain growth is affected.

## Conclusion

Brain development is a complex, multi-faceted process and this study explored just one aspect that is involved in fetal brain growth. A significant association between disruptions to the normal streaming of oxygen and nutrient-rich placental blood to the brain and reduced total and regional brain volumes in-utero in fetuses with CHD was identified. It was also observed that, when the fetal CHD results in a complete reversal in the normal streaming of fetal blood patterns, brain:body volume ratios are also reduced. In CHD subtypes where cerebral substrate delivery is expected to be normal, no significant volume differences were observed when compared to healthy control fetuses, highlighting the role of cerebral substrate delivery in fetal brain growth.

## Supplementary Material

Table S1

## Figures and Tables

**Table 1:** Classification of Congenital Heart Disease (CHD) According to Expected Cerebral Substrate Delivery in Study Population of 380 Fetuses

Group	N (M:F)	Median (IQR) GA at Scan (Weeks)
(0) Control	45 (22:23)	30.43 (29.43−31.14)
(1) Substrate content of cerebral blood is expected to be normal	210 (103:107)	31.71 (30.86−32.86)
(2) Substrate content of cerebral blood is expected to be mildly reduced	60 (33:27)	32.21 (31.25−33.14)
(3) Substrate content of cerebral blood is expected to be moderately reduced	45 (20:25)	32.29 (31.14−34.0)
(4) Substrate content of cerebral blood is expected to be severely reduced	20 (10:10)	33.43 (32.32−34.61)
TOTAL	380 (188:192)	31.71 (30.86−33.00)
