# Supplementary material for: Total and Regional Brain Volumes in Fetuses With Congenital Heart Disease
Source: J Magn Reson Imaging. Author manuscript; Available in PMC 2024 Aug 1. (PMC7616254; doi:10.1002/jmri.29078)
Supplement: Table S1 [file EMS197457-supplement-Table_S1.docx]

Supplementary material

| **Supplementary-Table 1:** A summary of all diagnoses included for this study, along with the rationale for inclusion in the categories described in Materials and Methods - Fetal CHD categorisation. N.B This is not an ‘exhaustive list’ of all possible CHD diagnoses, just those that were included in this study. The grouping/categorisation is not based solely on the underlying anatomy, and therefore minimises the need for speculation on cerebral substrate delivery. | | | |
| --- | --- | --- | --- |
| **Diagnosis** | **Potential (cerebrovascular) physiology associated with diagnosis** | **Factors considered when assigning to group** | **Rationale (Group)** |
| Normal (Control) | - | - | Normal cerebral substrate delivery (0) |
| Right aortic arch | Normal streaming, forward flow through left heart/aortic isthmus | Underlying anatomy | No anticipated effects of CHD on cerebral substrate delivery (1) |
| Double aortic arch | Normal streaming, forward flow through left heart/aortic isthmus | Underlying anatomy | No anticipated effects of CHD on cerebral substrate delivery (1) |
| Coarctation of the aorta, CoA (+) | Normal streaming, forward flow through left heart/aortic isthmus  Normal streaming, forward flow through left heart, reversal of flow at isthmus | Underlying anatomy; phase contrast flow; echocardiography | No anticipated effects of CHD on cerebral substrate delivery (1)  Mild reduction in cerebral substrate delivery (2) |
| Antenatally suspected coarctation of the aorta, not requiring surgery or intervention in the neonatal period, CoA (-) | Normal streaming, forward flow through left heart/aortic isthmus  Normal streaming, forward flow through left heart, reversal of flow at isthmus | Underlying anatomy; phase contrast flow; echocardiography | No anticipated effects of CHD on cerebral substrate delivery (1)  Mild reduction in cerebral substrate delivery (2) |
| Interrupted aortic arch | Normal streaming, forward flow through left heart to carotid arteries | Underlying anatomy | No anticipated effects of CHD on cerebral substrate delivery |
| Partial anomalous pulmonary venous drainage | Normal streaming, forward flow through left heart/aortic isthmus | Underlying anatomy | No anticipated effects of CHD on cerebral substrate delivery (1) |
| Ventricular septal defect | Normal streaming, forward flow through left heart/aortic isthmus | Underlying anatomy | No anticipated effects of CHD on cerebral substrate delivery (1) |
| Anomalous left subclavian artery | Normal streaming, forward flow through left heart/aortic isthmus | Underlying anatomy | No anticipated effects of CHD on cerebral substrate delivery (1) |
| Atrioventricular septal defect + coarctation of the aorta | Normal streaming, forward flow through left heart/aortic isthmus | Underlying anatomy | No anticipated effects of CHD on cerebral substrate delivery (1) |
| Dilated aorta | Normal streaming, forward flow through left heart/aortic isthmus | Underlying anatomy | No anticipated effects of CHD on cerebral substrate delivery (1) |
| Ventricular asymmetry | Normal streaming, forward flow through left heart/aortic isthmus | Underlying anatomy | No anticipated effects of CHD on cerebral substrate delivery (1) |
| Left atrial diverticulum | Normal streaming, forward flow through left heart/aortic isthmus | Underlying anatomy | No anticipated effects of CHD on cerebral substrate delivery (1) |
| Left atrial aneurysm | Normal streaming, forward flow through left heart/aortic isthmus | Underlying anatomy | No anticipated effects of CHD on cerebral substrate delivery (1) |
| Persistent left superior vena cava | Normal streaming, forward flow through left heart/aortic isthmus | Underlying anatomy | No anticipated effects of CHD on cerebral substrate delivery (1) |
| Cardiac apex to the right | Normal streaming, forward flow through left heart/aortic isthmus | Underlying anatomy | No anticipated effects of CHD on cerebral substrate delivery (1) |
| Mesocardia | Normal streaming, forward flow through left heart/aortic isthmus | Underlying anatomy | No anticipated effects of CHD on cerebral substrate delivery (1) |
| Cardiomegaly | Normal streaming, forward flow through left heart/aortic isthmus | Underlying anatomy | No anticipated effects of CHD on cerebral substrate delivery (1) |
| Cardiac mass | Normal streaming, forward flow through left heart/aortic isthmus | Underlying anatomy | No anticipated effects of CHD on cerebral substrate delivery (1) |
| Bilateral superior vena cava | Normal streaming, forward flow through left heart/aortic isthmus | Underlying anatomy | No anticipated effects of CHD on cerebral substrate delivery (1) |
| Total anomalous pulmonary venous drainage | Minimal if any effect on placental streaming, forward flow through left heart to carotid arteries | Underlying anatomy; phase contrast flow; echocardiography | No anticipated effects of CHD on cerebral substrate delivery (1) |
| Pulmonary Stenosis | Normal streaming, forward flow through left heart to carotid arteries (mild stenosis)    Reduced flow through the right heart with likely increased right to left atrial or ventricular shunting (moderate + stenosis + presence of VSD relevant). | Underlying anatomy; echocardiography | No anticipated effects of CHD on cerebral substrate delivery (1)  Mild reduction in cerebral substrate delivery (2) |
| Congenitally corrected TGA (ccTGA) | Normal streaming, forward flow through left heart to carotid arteries  Abnormal cardiac streaming, forward flow through left heart to carotid arteries  Normal streaming, forward flow through left heart, reversal of flow at isthmus | Underlying anatomy; echocardiography | No anticipated effects of CHD on cerebral substrate delivery (1)  Mild reduction in cerebral substrate delivery (2)  Mild reduction in cerebral substrate delivery (2) |
| Tetralogy of Fallot + absent pulmonary valve | Likely increased right to left atrial shunting | Underlying anatomy; phase contrast flow; echocardiography | Mild reduction in cerebral substrate delivery (2) |
| Aortic stenosis | Normal streaming, forward flow through left heart to carotid arteries  Reduced aortic flow with reversal at isthmus | Underlying anatomy; echocardiography | No anticipated effects of CHD on cerebral substrate delivery (1)  Mild reduction in cerebral substrate delivery (2) |
| Coarctation of the aorta with aortic stenosis | Normal streaming, forward flow through left heart to carotid arteries  Reduced aortic flow with reversal at isthmus | Underlying anatomy; phase contrast flow; echocardiography | Mild reduction in cerebral substrate delivery (2) |
| Unbalanced AVSD | Normal streaming, forward flow through left heart to carotid arteries  Reduced aortic flow with reversal at isthmus | Underlying anatomy; phase contrast flow; echocardiography | Mild reduction in cerebral substrate delivery (2) |
| Tetralogy of Fallot (including double outlet right ventricle) | Reduced flow through right heart with increased right to left atrial or ventricular shunting | Underlying anatomy | Mild reduction in cerebral substrate delivery (2) |
| Tricuspid dysplasia | Reduced flow through right heart with increased right to left atrial shunting | Underlying anatomy | Mild reduction in cerebral substrate delivery (2) |
| Common arterial trunk | Complete admixture of placental and fetal systemic venous blood | Underlying anatomy | Moderately reduced SaO2 in carotid arteries (3) |
| Hypoplastic left heart syndrome | Complete admixture of placental and fetal systemic venous blood | Underlying anatomy | Moderately reduced SaO2 in carotid arteries (3) |
| Pulmonary Atresia | Complete admixture of placental and fetal systemic venous blood | Underlying anatomy | Moderately reduced SaO2 in carotid arteries (3) |
| Hypoplastic right heart syndrome | Complete admixture of placental and fetal systemic venous blood | Underlying anatomy | Moderately reduced SaO2 in carotid arteries (3) |
| Double outlet right ventricle + mitral atresia | Complete admixture of placental and fetal systemic venous blood | Underlying anatomy | Moderately reduced SaO2 in carotid arteries (3) |
| Double outlet right ventricle + pulmonary atresia | Complete admixture of placental and fetal systemic venous blood | Underlying anatomy | Moderately reduced SaO2 in carotid arteries (3) |
| Critical aortic stenosis with intact atrial septum. | Complete admixture of placental and fetal systemic venous blood | Underlying anatomy | Moderately reduced SaO2 in carotid arteries (3) |
| Transposition of the great arteries + double outlet right ventricle + coarctation of the aorta | Reversal of normal fetal streaming with forward flow at the aortic isthmus | Underlying anatomy | Severely reduced SaO2 in carotid arteries (4) |
| Transposition of the great arteries | Reversal of normal fetal streaming | Underlying anatomy | Severely reduced SaO2 in carotid arteries (4) |
